# Operations on the Kidney

**Published:** 1893-01

**Authors:** Herman Mynter

**Affiliations:** Buffalo, N. Y.; Professor of Operative and Clinical Surgery, Medical Department, Niagara University; 195 Franklin Street


### OPERATIONS ON THE KIDNEY.[1]

By HERMAN MYNTER, M. D., Buffalo, N. Y.

Professor of Operative and Clinical Surgery, Medical Department, Niagara University.

During the last few years, I have had the opportunity of operating on the kidney ten times, two operations being done on one patient. I might add an eleventh case operated upon a number of years ago for stone in the kidney. Three of these operations were nephrorraphies for floating kidneys, three nephrotomies for pyonephrosis, two nephrectomies,—one for sarcoma of the kidney, and one for tuberculosis of the kidney,—one a nephrotomy for a crushed kidney, one a nephrolithotomy, and, lastly, one operation was done for a supposed cholelithiasis and a floating kidney found. Although this number of cases is small, especially when compared with those in the larger clinics, it is sufficiently large and varied to gain some experience in this field of surgery. Ten of the eleven cases recovered from the operation, although one of them died four weeks later of pneumonia, and one died of suppression of urine following nephrectomy. In the short time at my disposal tonight, I cannot, of course, go over all the different indications for operations on the kidney and the different methods. I shall, therefore, confine myself, first, to relating the histories, and, secondly, to discussing such points which, in my estimation, are of superior importance :

Case I.—*Nephrorraphy for Floating Kidney.*—Mrs. E., 51 years of age, entered the Sisters' Hospital in December, 1890. When eighteen years of age, she noticed pain in the region of the right kidney. Nevertheless, she had no distressing symptoms, till three years previously, when the pain increased and became more or less permanent. At the same time she commenced to complain of dysuria and, occasionally, of bloody urine. During the last year she had complained of dyspeptic phenomena, had become extremely nervous and hysterical, so much so that her family considered her insane, and avoided her as much as possible. At the examination she looked thin, emaciated, and presented many symptoms of acute melancholia. She complained especially of pain over the right inguinal region. An oval tumor with all the characteristics of the kidney was felt here. It was very movable, could be grabbed through the thin abdominal wall, and moved up and down, and in and out. The urine was normal and the examination gave otherwise negative result. December 24, 1890, nephrorraphy was done by the usual oblique incision along the outer margin of the quadratus lumborum muscle, the kidney was found very

1. Read at the Stated Meeting of Buffalo Academy of Medicine, December, 6, 1892.

movable, moving up and down during respiration. Palpation did not show anything abnormal. The fibrous capsule was opened behind and stripped off for about two square inches, and the kidney fastened by four silkworm-gut sutures, two on each side, which passed deeply through the kidney substance, the fibrous capsule, and the fascia and muscles. The wound was packed with iodoform gauze down to the kidney itself. The first dressing was allowed to remain in place for fourteen days, and the wound was thereafter dressed in a similar way once a week. She was kept in bed six weeks, and then left the hospital with the wound healed. The kidney was then immovably fixed to the posterior wall. She was a great deal less tender from pressure; she had gained in weight, but her hysterical symptoms had not improved. I have not heard from her since.

Case II.—*Nephrorraphy for Floating Kidney.*—Mrs. P., aged 39, wife of a physician, entered Sisters' Hospital on August 25, 1892. She had complained of pain in the right side for four years, occurring after confinement. The pain was steady and the whole region so tender, that she could not stand the slightest pressure, and no examination could be made. The pain was accompanied with a severe dragging feeling and was increased by the slightest exertion. Her general health had commenced to suffer; she was thin, extremely nervous, complained of insomnia and inability to do anything whatever. She had consulted many physicians. One ascribed her symptoms to a lacerated cervix and operated it; another treated her for a prolapsed womb; a third declared she suffered from chronic ureteritis, etc. Menstruation was regular, walking almost impossible on account of pain. Under narcosis I discovered a prolapsed and movable kidney in the tender region. As all her symptoms pointed to this region and probably were dependent upon this prolapsed kidney, nephrorraphy was performed on August 6, 1892, in a similar way, only that the fibrous capsule was stripped off from the whole posterior surface, and that six kangaroo tendons were used as sutures.

The after-treatment was the same, and she left for her home in eight weeks. She had gained some in weight, could sleep better, and was less nervous and hysterical, had a good deal less pain, and could stand considerable pressure in the right inguinal region. Nevertheless, the result has, so far, not been as perfect as had been hoped for. She still complains of sleeplessness, and has some tenderness in the region.

Case III.—*Nephrorraphy for Floating Kidney.*—Mr. F., aged 32, entered the hospital on September 8, 1892. He had for seven years complained of indigestion. Two years ago, while lifting a heavy weight, he felt "something give away," had considerable pain, but did not pay any particular attention to it till six months ago, when he felt a tumor in the right side of the abdomen. During the year previously, he had complained of a dull heavy feeling in the right inguinal region, occasionally in the left inguinal region, too. During the same time, his family observed increasing nervousness and irritability, verging on insanity ; he was fretful, peevish, lost in health and weight, and was unable to work. By examination with Dr. Fowler, a tender and movable lump was felt in the right inguinal region, having all the characteristics of the kidney. Left kidney was also prolapsed, although not in so high degree, and was somewhat tender.

September 8, 1892, nephrerraphy on right kidney, which was fastened to the posterior wall by six kangaroo tendons, the fibrous capsule having first been stripped off. It was quite difficult to find the kidney and bring it up into the wound, it having fallen far into the abdominal cavity, and we succeeded only by gradually pulling the fatty capsule out with artery forceps and cutting it away.

The after-treatment was conducted on similar lines as in the other two cases. He was kept in bed eight weeks ; the wound is healed, the tenderness considerably decreased, the appetite good, the weight increased, but he is still peevish, fretful, and hypochondriac, and spends most of his time in bed. Time alone will show whether the operation will benefit him permanently or not.

CASE IV.—*Nephrotomy for Pyonephrosis.*—Mrs. M. K., aged 35, entered the hospital on December 10, 1890. She had for about one year complained of severe pain in the right lumbar region, coming on periodically every ten or fourteen days. The pain would occur on rising in the morning, and last till about noon, when she would pass a large amount of urine and pus with immediate relief. She would then feel well for a week or two, when a similar attack would occur. Half a year ago a tumor was discovered in the right lumbar region. It was at the examination of the size of the head of a man, filling out the whole space between the ribs and the ileum and extending forward to the outer margin of the rectus muscle. A distinct deep fluctuation could be felt both anteriorly and posteriorly. The urine was torpid and contained considerable pus.

On December 12th, nephrotomy—a puncture with a hypodermic needle having previously revealed pus. The incision was made along the outer margin of the quadratus lumborum muscle, the kidney opened, and about three pints of pure yellow pus removed. The interior of the kidney felt like one large pus sac, and no kidney tissue could be felt.

The cavity was irrigated with corrosive sublimate, the sac stitched to the skin, two large drainage tubes introduced, and antiseptic dressings applied. The sac contracted rapidly, and the patient left on January 6, 1892, with the wound almost healed, except a very small fistule through which a little pus was discharged. I have not heard from her since.

Case V.—*Nephrotomy for pyonephrosis.*—G. P., aged 37, entered the Hospital on September 6, 1891. He had emaciated steadily for two years and complained of frequent urination. He knew of no cause, had received no injury, and his condition had not been diagnosticated till Dr. Cronyn saw him a few days previously. A large fluctuating swelling was felt over the left kidney, very similar to that of the previous case. The urine contained a large amount of pus. By posterior incision one quart of pus was evacuated, the cavity drained and irrigated. He left the Hospital greatly improved in six weeks, but with a fistule discharging some pus. I have not heard from him since.

Case VI.—*Nephrolithotomy.*—Mrs. D., 45 years of age, had complained for two years of dysuria with bloody urine and pain in left lumbar region. The pain increased gradually, so that she was confined to her bed and had to use morphine continually. In January, '79, a tumor was discovered in the left lumbar region. It was punctured in March, 1879, and four ounces of pus evacuated. The swelling increased again and perforated at the point of puncture, leaving a fistule behind, through which stones occasionally were discharged. The urine contained considerable pus. The operation was done on December 31, 1879. To give place, two inches of eleventh rib were resected, but all the tissues were changed into a fibrous, almost cartilaginous, unyielding tissue, and no trace was left of the normal anatomy. The kidney could not be distinguished as such, the margin of the quadratus lumborum being the only thing which could positively be recognized. A number of stones were removed from a cavity in the supposed kidney, but nephrectomy, which I intended to make, had to be given up. She died four weeks later of pneumonia. Even at the post mortem it was found impossible to separate the kidney. It was intimately connected with the pancreas and colon descendens, as large as a fist, and contained a great number of cavities filled with pus and stones.

Case VII.—*Nephrectomy.*—Mrs. S., 53 years of age, entered the Sisters' Hospital on October 14, 1892. She fell down stairs three years ago ; one month later she commenced to complain of pain in left lumbar region, extending down into the thigh, and bloody urine. The pain and bleeding had since recurred periodically, especially when she walked around. Occasionally, she had passed clotted blood and had then had a good deal of dysuria. At present she complained of a deep-seated, boring, and gnawing pain in the left lumbar region, and of frequent micturition of bloody urine with a great deal of dysuria. She was referred to me by Dr. Cronyn, with the probable diagnosis of cancer of the kidney. A large movable tumor could be felt in the left lumbar region, extending forward almost to the outer margin of the rectus muscle ; the tumor felt hard and solid. She had lately commenced to lose flesh, and had a slight yellowish color of the skin. The amount of urine for three days averaged sixty ounces, containing considerable blood and traces of albumen.

October 17, 1892, nephrectomy by König's lumbo-abdominal incision, being a longitudinal incision along the margin of the quadratus lumborum muscle, and curving forward from its lower end along the crista ilei well towards the outer margin of the rectus muscle. Without much difficulty the large tumor was isolated, separated and brought up into the wound. The pedicle was perforated from behind with a blunt aneurism needle near the kidney and doubly ligated with chromated catgut. As additional security, a pair of large curved forceps were applied outside the ligature, and the kidney then removed. The enormous wound was plugged with eighteen yards of iodoform gauze strips, and the incisions closed with sutures except two inches behind.

The further result was extremely favorable; the temperature rose once to 102°, but was normal from the fifth day. The amount of urine on first and second days was ten ounces to sixteen ounces, third day, possibly on account of the use of digitalis, sixty ounces, and since that time about sixty ounces every day; the urine became in a few days normal. The wound healed kindly and the patient left the hospital in four weeks recovered.

I add the examination of the kidney by Dr. Wm. C. Krauss: Weight of kidney, fifteen ounces; size, greatest length, sixteen centimeters; greatest width, eleven centimeters; greatest thickness, eight and one-half centimeters; shape — the kidney presents an irregular unsymmetrical appearance, possessing little of its original kidney shape. Its predominant characteristic is the number of large nodules which are implanted upon its surface and which seem, in fact, to compose the entire organ. The cephalic end is somewhat tapering, while the caudal end is broad, bifurcated, and massive. On its convex surface a large flattened nodule is found growing like a cauliflower excrescence, and separated from the caudal and cephalic nodules by deep furrows. The body of the kidney presents a multi-nodular appearance with shallow, winding grooves. The concavity of the kidney has little to warrant its name, being more convex than concave. A large mass of fat found here renders it difficult to dissect out the ureter and the renal vessels. The consistency of the mass varies. The nodules at the cephalic end and the one on the convex surface are soft and fluctuating, while the body of the kidney and the nodules at the caudal end are hard, dense, and firm. The capsule was non-adherent, not thickened, and easily torn.

On section, nothing could be found of the pelvis, and the cut surfaces show large alveoli filled with a soft, reddish grumous mass. Hemorrhages had taken place in some of these alveoli. A small section of the caudal nodules still retains some kidney structure, but with this exception the entire organ is transformed into diseased tissue. Examining some of the soft, semi-fluid matter microscopically, revealed a predominance of small, round cells, some small spindle cells, and a minimum amount of inter-cellular tissue. These cells are characteristic of sarcomatous growths, and decide the nature of the tumor, namely, a small round-celled sarcoma.

The external appearance of the kidney, presence of large alveoli, and clinical history, are all in harmony with the diagnosis.

CASE VIII.—*Nephrectomy following nephrotomy.*—Miss S., aged 24, had for several years complained of pains in left lumbar region. The attacks became more and more frequent, and, at last, almost continuous. During the last half-year she had had painful and frequent micturition. The urine contained a great amount of pus, but was, at times, almost clear. By the examination with Dr. Thornton, a tumor was felt at the left kidney, very sensitive to pressure. The kidney could be felt by bi-manual examination, generally enlarged, and could be easily palpated through lumbar region.

An explorative incision was made in February, 1891, and about two ounces of pus removed from the pelvis of left kidney. The wound did not heal, and took on a tuberculous appearance, but the pain was very much relieved, as was the dysuria. Ten weeks later, in May, 1891, the kidney was removed by nephrectomy and found to be tuberculous throughout. Total suppression of urine followed, and she died of uremia four days later. *Post-mortem* examination was made, but so late that nothing could be decided in regard to the other kidney.

CASE IX.—*Nephrotomy for Crushed Kidney.*—In January, 1891, I was called to Oil City, Pa., to see Mr. W., aged 22, who, four days previously, had been caught obliquely between the bumpers while coupling cars. He was able to travel thirty miles after the accident, but complained then of faintness, pain, vomiting, and dysuria. The water contained a good deal of blood, tympanites supervened, and the temperature increased. It was supposed by his three attending physicians that he had either ruptured his guts or bladder, and that a laparatomy was necessary. At the examination profuse ecchymosis was seen around the anus and perineum, the urine was of dark, dirty color, intensely mixed with blood, a fulness and diffuse ecchymosis were seen in right lumbar region. As the diagnosis of crushed kidney seemed evident, the patient was removed to Buffalo the same night, and nephrotomy performed right away on his arrival. The kidney was found lying in a large cavity filled with blood and urine, and the lower half was crushed to a pulp. The bleeding was so terrific that nephrectomy could not be done. I, therefore, simply disinfected the wound and plugged the whole cavity with iodoform gauze. The urine passed for a time through the wound, the crushed parts came away by irrigation, and he left the hospital recovered in eight weeks. He was killed on the same railroad half a year later.

The following case is of interest on account of the wrong diagnosis:

Case X.—Mrs. R., 45 years of age, had for six months complained of severe pain one and a half inches to the right of the ensiform cartilage. The pain would come periodically, and be attended with yellow color of skin and conjunctivæ and clay-formed stools. Five weeks previous to her entrance in the hospital, she had symptoms indicating peritonitis, followed by a very severe attack of pain and jaundice, lasting four days. She felt then a lump in right hypochondriac region of the size of an egg, and corresponding to the gall-bladder. She entered in order to have a cholecystotomy performed, it being supposed from the history and symptoms that she suffered from gall-stone colic. Over the region of the gall-bladder a pear-formed, nodulated, apparently immovable tumor was felt, which had all the appearances of an indurated gall-bladder filled with gall-stones.

An explorative incision was made in September, 1892, along the right margin of the rectus muscle, but on entering the abdominal cavity, much to our surprise, no tumor was found, and even the gall-bladder was absent. A hard lump was felt behind the colon transversum. An opening having been torn through the mesentery of the colon transversum, it was found to be a floating kidney. The wound was, therefore, closed, and the patient left the hospital in two weeks. She has felt well since, and, so far, had no attacks.

A similar mistake is reported by Dr. Willy Meyer, who, also, opened the abdomen for supposed tumor of the gall-bladder, but found a chronic purulent nephritis and pyonephrosis, which, twelve days later, was removed by nephrectomy. Thirty-eight days later, during menstruation, sudden suppression of the urine occurred, for which, two and a half days later, nephrotomy was performed on the only remaining kidney, and the ureter, for two inches, found blocked with shreds, blood and pus. They were removed, the ureter flushed out, and the kidney at once resumed its function, and the renal fistula healed in the course of eight weeks.

This case is of interest in suggesting a possible help in case of total anuria, following a nephrectomy. I have little doubt but that nephrotomy ought to have been done in my fatal case of nephrectomy. Similar successful operations of nephrotomy for suppression of urine after nephrectomy have been reported by Bardenheuer, Lange, Clement, Lucas, and others.

If, as Willy Meyer pertinently asks, suppression sets in immediately after a nephrectomy, where the retroperitoneal incision has been made, where strong antiseptic solutions have not been used, where there has been no shock and no great loss of blood, where the cystoscope has shown a well-working kidney on the other side, and where continuous stimulation fails to work—is not, then, nephrotomy on the other side indicated, as it is the only remaining hope? If no obstruction is found, artificial depletion of the hyperemic organ may, perhaps, prove of value.

Floating kidney may exist without "special symptoms. On the other hand, through the severity of pain, the inability to work, the gastric and mental complications, and the disturbances in nutrition, life may be materially shortened. The dragging pain predisposes to strange fits of melancholia, there is almost always loss of appetite, with vomiting and cardiac palpitations. The neuralgic pain is always on the affected side, and, at times, most excruciating in character." (*Med. Record*). The vomiting may increase to complete gastric intolerance (Mathieu), and be accompanied with, or terminate in, severe gastralgic pains. The vomiting is not preceded by nausea. The cause is, according to Mathieu, traction on the duodenum or peritoneum. Careful search should be made for floating kidney, where vomiting constitutes the principal symptom, as, otherwise, such vomiting may suggest tabes, pregnancy, gastric ulcers, intestinal obstruction, hysteria, or poisoning.

Oerum mentions as etiology of floating kidney, abnormal length of renal vessels and the presence of a mesentery, but cannot determine whether it is congenital, or acquired little by little.

Bang, of Copenhagen, found in two post-mortems, that the floating kidney was detached from the suprarenal gland, which kept its place, that the fat in the capsule had disappeared, and that the branches from the renal artery to the suprarenal gland were wanting. He mentions that floating kidney seldom is found on the left side, and gives, as cause, that the left suprarenal veins empty into the renal vein, while on the right side they empty directly into vena cava. This seems to confirm his idea about the detachment of the suprarenal gland. He explains the symptoms, pain, vomiting, chills, and diminished diuresis, as an acute retention of urine, and acute hydronephrosis, on account of sudden dislocation of the kidney, by which the upper part of the ureter is so much bent that the urine cannot escape. When the hydronephrosis has reached a certain degree, the pressure will surmount the difficulty. Mathieu explains the symptoms in a similar way.

Dr. Keen has collected 134 cases of nephrorraphy with four deaths, *i. e.*, 2.9 per cent.

Of 116 cases, reviewed after at least three months had passed, 57.8 per cent. were cured, 12.9 per cent. improved, and 19.8 failed. When the fatty capsule alone was sutured, the failures amounted to 26.6 per cent. If the fibrous capsule was sutured, 25.9 per cent. were failures; if the kidney itself was included in the suture, 13.5 per cent. were failures.

Prof. Riedel thinks that the reason why so many operations for floating kidney have been unsuccessful, is that the organ, soon after the operation, changes its position, its lower portion turning forwards and lying upon the inner surface of the posterior upper margin of the pelvis. This may be avoided if the kidney is also attached to the diaphragm. Riedel tried to accomplish this in three cases by suturing the lower portion of the kidney to the periosteum of the twelfth rib. It was unsuccessful, because the upper portion of the organ was displaced forwards and the cicatrix became painful. He has since, in five cases, used another method successfully, and the patients have kept well from one to one and a half years. He exposes the kidney by the usual incision, opens the fatty and fibrous capsule, and pushes, then, the organ so far upward behind the diaphragm, that only its lower half is exposed to view. The median portion of the fibrous capsule is then fixed to the anterior portion of the quadratus lumborum muscle by deep catgut sutures, while at the sides the peritoneum and subserous fat is marginated and sutured to the capsule. In this way the lower segment is fixed, while the upper portion is still detached and lies upon the diaphragm. A strip of iodoform gauze is now thrust up between the kidney and the diaphragm, so that the entire posterior surface of the kidney is covered. A second gauze tampon is introduced into the place formerly occupied by the kidney, and a third is placed upon the lower portion of the organ, lying upon the anterior surface of the quadratus lumborum muscle. To each strip a strong silk thread is attached. The sacro-lumbalis and abdominal muscles are sutured over the tampons, leaving openings for their extraction. The cutaneous wound is thereafter plugged. The tampons are withdrawn in four weeks, unless necessary earlier; first, the middle one, then the lower one, and then the upper one, and a deep cavity is then found lined with granulations. A drainage-tube is now introduced, and the wound heals rapidly. The idea is, of course, to produce strong adhesions to the diaphragm. The patients were kept quiet in bed for twelve weeks.

In regard to the mortality after nephrectomy, it deserves mention that Schede has extirpated the kidney seventeen times with one death. This is a different death percentage than usually given. Gross (223 cases) gave a death-rate of 44.6 per cent., Broden, of Paris, (235 cases,) 44.4 per cent., Czerney (72 cases), 44.4 per cent. Newman gives the mortality, after lumbar nephrectomy, as 30.5 per cent., after abdominal nephrectomy, 47.1 per cent. Schede considers the retroperitoneal transverse section in itself an almost harmless method. In cases of exhaustive hemorrhage from the kidney, nephrectomy is the only remedy. In one interesting case of renal hemorrhage, in which it was doubtful which kidney was affected, Schede opened the bladder by suprapubic cystotomy, inserted catheters into both ureters, and brought the ends out through the penis by aid of a lithoclast. The bladder wound was thereafter closed. The same evening the blood was seen to escape from the catheter in the left ureter.

The question about healing of wounds in the kidney and regeneration of renal tissue after wounds, has been clearly answered by Barth, of Marburg. After a wound in the kidney, healing occurs in the usual way. A bloodclot fills the defect, connective tissue proliferation takes place, and, lastly, cicatricial contraction occurs, but the cicatrix is never filled with glandular structure again, and there is no such a thing as a re-creation of new and active renal tissue. A compensatory hypertrophy in the intact portion of the operated kidney, as well as in the other kidney, may, perhaps, occur, the glomeruli enlarge, the capsules dilate, and the canaliculi grow in circumference and length. These changes are observable in the cortical substance by an increase in size of all its elements.

Two instruments are of importance for diagnostic purposes, particularly in nephrectomy. It is of the utmost importance to find out whether the other kidney is in good working order or not. Catheterizing the ureter is in the female, at best, a very difficult proceeding; in the male, impossible without first performing perineal cystotomy, or else, as Schede did, suprapubic cystotomy. By the use of Leiter's cystoscope with incandescent lamp, we are able to see the openings of the ureters, the jets of urine as they pass out through the ureters, to note their frequency, color, and consistency. With a common urethroscope with electric or simply reflected light, we may probably be able to see the ureters, if introduced into the bladder through a perineal wound in the male or through the urethra in the female. The cystoscope has, probably, a great future, both in regard to diseases of bladder and of kidneys.

Fenwick has published his experiences with the cystoscope in the *British Medical Journal* of October 18, 1890. He recognizes the dangers of performing nephrectomy without investigating the working capacity of the other kidney. Cystoscopy of the urethral orifices is a great help in this direction. The efflux of urine establishes at once the presence of a working kidney. The sluggishness or rapidity with which the jets succeed each other mark the absence or presence of irritation in the pelvis. The color of the jet, whether it be clear, muddy, or bloody, testifies to the normal or abnormal condition of the secretion. The urethral orifice, moreover, becomes implicated by descending changes from the pelvis of the kidney, and its shape and appearance may indicate a corresponding condition in the pelvis and ureter, particularly in scrofulosis.

Three methods have been used for nephrectomy : (1) Abdominal nephrectomy, through Langenbeck's incision in the semilunar line, or through Barker's median incision ; (2) lumbar nephrectomy by oblique incision along the outer margin of the quadratus lumborum muscle, the incision being enlarged by curving forwards from its lower end towards the umbilicus (König's method), or by adding a vertical incision downwards from the upper end of the oblique incision (Moris's method) ; (3) by lateral extraperitoneal incision, reaching from the eighth rib to the anterior superior spine of the ileum. The different muscular layers are cut through and the peritoneum stripped up from the iliac fossa and from the anterior suface of the kidney. If we compare these three methods, there is little doubt but that the lumbar method is the most advantageous, particularly König's. Excellent space is gained and the kidney may be exposed with great facility, the peritoneum is not opened, and good drainage is provided in the proper place. The abdominal nephrectomy has but one advantage, *i. e.*, that we may satisfy ourselves by palpation of the condition of the other kidney before proceeding to remove the diseased one, and this advantage is more than counterbalanced by the dangers incidental to a laparatomy.

The lateral extraperitoneal method offers no advantages over the lumbar incision, and predisposes more to ventral hernia. The question, whether nephrectomy ought to be performed primarily for a large pyonephrosis or only secondarily following a nephrotomy, is not settled. In my cases I chose nephrotomy, sewing the sac to the skin, and I doubt very much that primary nephrectomy can be done in cases in which the kidney has been transformed into a large thin-walled sac containing more than a quart of pus. I should consider it advisable in such cases to wait with the nephrectomy until the sac has contracted. Even then the nephrectomy will probably be difficult on account of adhesions between the peritoneum and the former cyst wall.

One question more deserves a few words, Where to incise the kidney in cases of nephrolithiasis, and for other explorative purposes? Mr. Jordan Lloyd has proposed to make a puncture in the lower end and posterior border of the kidney with a long-bladed tenotome, in the direction upwards and inwards, making for the lowest of the calyces, and through this opening introduce a proper sound and systematically explore the pelvis and calyces. Mr. Moris advises to enlarge this wound sufficiently to introduce the finger and instruments. There is, of course, more danger of hemorrhage by this proceeding, but, on the other hand, Barth has shown that such wounds may be sutured and heal with ease, and the hemorrhage will, probably, stop by a properly applied suture. Secondly, we avoid, probably, the urinary fistula, which is apt to occur if we make our incision into the posterior wall of the pelvis. Mr. Thornton maintains, in opposition to most other writers, that wounds into the pelvis of the kidney heal well enough. I have no particular experience upon this point, as all my nephrotomies were made for pyonephrosis, in which the whole kidney was changed into a pus-sac, with the exception of one case—number eight—in which nephrotomy was done for tuberculous kidney with moderate pyonephrosis. Urinary fistula followed, as a matter of course, and nephrectomy was done ten weeks later, resulting in death from anuria. Nevertheless, we could only think of incising the kidney through its substance, when we felt sure of the diagnosis—stone—and were able to suture the wound again. If the kidney has to be drained, the pelvis is the only proper spot for incision.

195 Franklin Street.

---

"Are there too many doctors?" asks an exchange.

"No, there are not half enough; but there are too many men pretending to be doctors who are not."—*Texas Siftings.*

